# Immunodeficiency in DiGeorge Syndrome and Options for Treating Cases with Complete Athymia

**DOI:** 10.3389/fimmu.2013.00322

**Published:** 2013-10-31

**Authors:** E. Graham Davies

**Affiliations:** ^1^Centre for Immunodeficiency, Institute of Child Health, University College London and Great Ormond Street Hospital, London, UK

**Keywords:** DiGeorge syndrome, immunodeficiency, thymus transplantation, 22q11 deletion, T-cell development

## Abstract

The commonest association of thymic stromal deficiency resulting in T-cell immunodeficiency is the DiGeorge syndrome (DGS). This results from abnormal development of the third and fourth pharyngeal arches and is most commonly associated with a microdeletion at chromosome 22q11 though other genetic and non-genetic causes have been described. The immunological competence of affected individuals is highly variable, ranging from normal to a severe combined immunodeficiency when there is complete athymia. In the most severe group, correction of the immunodeficiency can be achieved using thymus allografts which can support thymopoiesis even in the absence of donor-recipient matching at the major histocompatibility loci. This review focuses on the causes of DGS, the immunological features of the disorder, and the approaches to correction of the immunodeficiency including the use of thymus transplantation.

## Introduction

DiGeorge syndrome (DGS) was first described in the 1960’s and classically comprises T-cell deficiency (due to thymic hypoplasia), hypoparathyroidism, cardiac malformations, and facial abnormalities. Subsequently, it was recognized that deletions of the long arm of chromosome 22 at position q.11 were most commonly associated with DGS (1, 2). DGS is also found associated with other genetic abnormalities and with certain teratogenic influences. It was also recognized that multiple other clinical features could be associated with this deletion. The DGS phenotype is very heterogenous with variable expression of the different features including the immunodeficiency.

## Causes of DGS

### Early thymic development

At an early stage of embryonic development the pharyngeal apparatus can be recognized. This becomes segmented into a series of pharyngeal arches and pouches each comprising an outer ectodermal and inner endodermal layer separated by mesodermal tissue and neural crest cells (NCC) (3, 4). The thymus, parathyroid glands and great vessels of the heart develop from these structures notably the third and fourth arch structures. Thymic epithelial development is under the control of the transcription factor, FoxN1, and studies of expression of this factor have demonstrated that the thymus derives from an area of the endoderm in the ventral aspect of the third pouch (5). The mesoderm and NCC contribute to the thymic connective tissue including vascular endothelium and mesenchymal cells, the latter thought to be important in regulating early thymic epithelial development (6). Parathyroid gland development is closely allied, this organ being derived from the endoderm of the ventral part of the third pharyngeal pouch again with mesodermal cells and NCC contributing the connective tissue and vascular endothelium. From the eighth week of human gestation, bone marrow derived T-cell precursors have been shown to enter the thymic structure (7). The further development of the thymus is dependent on two-way interactions between these lymphoid cells and the thymic stroma (8, 9).

Hematopoietic cell defects resulting in severe combined immunodeficiencies lead to failure or disturbed thymic development as a consequence of failure of this lymphoid – stromal interaction (10, 11) which can be reversed by successful hematopoietic stem cell transplantation (12). These aspects of thymic stromal deficiency are considered elsewhere in this Research Topic.

The classical features of DGS occur as a result of the early embryonic disturbance of development of the pharyngeal arch apparatus and are independent of the influence of hematopoietic cell precursors on thymic development.

### Genetic associations of DGS

DiGeorge syndrome overlaps considerably with velocardiofacial (VCF) syndrome and to a lesser extent with conotruncal anomaly face syndrome; all these are associated with hemizygous 22q.11 deletions manifesting with a wide array of clinical features (13). The deletion is also associated with neurodevelopmental delay, behavioral, and psychiatric features. The multitude of possible clinical features (over 180) have been reviewed by Shprintzen (14). DGS and VCF are sometimes collectively referred to as the 22q.11 deletion syndrome. The incidence of this deletion is high at around 1:4000 (15). In 90–95% of cases this arises *de novo* with the other 5–10% being inherited from an affected parent (13). Over 90% of cases have a typical 3 Mb deletion including over 30 different genes (16). This seems to occur between two regions with homologous low copy repeats suggesting that deletion occurs through a process of homologous recombination. Most other patients have a smaller, 1.5 Mb, deletion (17, 18). There is no correlation between the size of the deletion and the clinical phenotype. Discordance between phenotypes has been described in monozygotic twins carrying the deletion (19). In rare cases mutations in a single gene, TBX1, have been described resulting in the DGS phenotype (20, 21). TBX1 is one of the T-box genes with an important role in regulating the expression of transcription factors (22). Studies of a mouse model with a syngenic deletion on chromosome 16 have helped elucidate the role of Tbx1. Homozygous deletions of this gene result in a very severe, lethal phenotype including all the features of DGS whilst hemizygous loss of the gene produces a milder phenotype with variable penetrance of the different clinical features (23). However, implicating TBX1 as the sole gene causing DGS in 22q deletion syndromes may not be the whole story. Adjacent deletions not involving TBX1 can give a phenotype with some overlapping features (24) as can atypical deletions covering different critical regions in the same part of the chromosome (25). Other genes in the region, also affected in the typical DGS deletion, may have a modifying effect on expression of the disorder. These include CRKL, coding for an adaptor protein involved in growth factor signaling. Crkl is expressed in neural crest derived tissues and in mice null for the gene there is aberrant or absent thymic development (26). However, hemizygous Crkl loss is not associated with an abnormal clinical phenotype suggesting a gene dosing effect. The effect of compound heterozygosity for Tbx1 and Crkl deletions, on development of DGS features, is additive (27). The function of TBX1 is complex and mediated through regulation of downstream transcription factors. The detailed role of TBX1 in 22q.11 deletion syndromes and in thymus development in particular has been reviewed by others (28, 29).

A much rarer but well characterized genetic association with a DGS phenotype occurs with interstitial deletions at chromosome 10p (30–33). This has been designated DGS 2.The clinical phenotype overlaps with that associated with 22q.11 deletion but with some important differences. Sensorineural hearing loss and mental retardation are relatively common features in those with 10p deletions but rare in 22q11 deletion cases; renal anomalies, and general growth retardation are more prevalent in 10p deletion than in 22q11 deletion cases (34). Deletions at 10p syndrome have been estimated as having an incidence of 1 in 200,000, some 50 times less common than 22q.11 deletions (35, 36). The role of the genes deleted and responsible for the clinical picture is less well understood than in 22q deletion DGS but on-going work has identified some critical regions involved in developmental abnormalities (32, 37).

Mutations in the Chromodomain Helicase DNA-binding protein 7 (CHD7) gene are responsible for most cases of Colobomata, Heart defect, Atresia choanae, Retarded growth and development, Genital hypoplasia, Ear anomalies/deafness (CHARGE) syndrome. A DGS phenotype including complete athymia may be part of this syndrome but there is marked variability in expression of the multiple clinical features. The incidence has been estimated at 1 in 8500 (38). CHD7 acts as a regulator of transcription of other genes. Its expression has been demonstrated in the NCC of the pharyngeal arches. Normal development of these structures has been shown to be dependent on the co-expression of Chd7 and Tbx1 in mice suggesting the likely mechanism by which CHARGE syndrome can lead to a DGS phenotype (39, 40).

### Non-genetic associations of DGS

Embryopathy induced by exposure of the fetus to retinoic acid can include a DGS phenotype (41). Retinoic acid affects Tbx1 expression in avian embryos (42) whilst it has also been shown that Tbx1 can, in at least some circumstances, regulate retinoic acid metabolism (43). Fetal alcohol syndrome (44–46) and maternal diabetes (47, 48) have also been associated with the DGS phenotype. In the latter, there is often an associated renal agenesis. It has been postulated that maternal diabetes can lead to interference with neural crest and mesenchymal cell migration (49).

## Immunological Features of DGS

### Incidence and severity

DiGeorge syndrome may be associated with a complete range of T-cell deficiency from normal T-cell numbers and function to complete DGS (cDGS) with a T-negative severe combined immunodeficiency (SCID)-like picture. It was recognized early on that the T-cell immunodeficiency may be incomplete and the term partial DGS (pDGS) was coined (50). In a large series of patients with 22q11 deletions, the proportion of affected individuals falling into the cDGS category was around 1.5% of the 218 who underwent immunological testing or around 0.5% of the whole series of over 550 patients (13). A much higher proportion had minor laboratory abnormalities suggesting pDGS. In one series, from a major referral center, mild-moderate lymphopenia, consistent with pDGS, was reported in 30% of 22q.11 patients (51).

Less is known of the frequency of severe immunodeficiency in 10p deletion DGS. A review of published cases identified low levels of T cells and immunoglobulins as well as a small or hypoplastic thymus in 9 of 32 (28%) patients evaluated. However none of these patients were reported as having significant infections, suggesting that the immunodeficiency was likely partial rather than complete (34).

In CHARGE syndrome, severe immunodeficiency has been described (51–55). The proportion of cases affected with immunodeficiency is not well established as there is no reported large series looking at immunological parameters. Immunodeficiency may not always be considered in CHARGE; one recent report of a large series of 280 cases did not provide any information on the prevalence of recurrent infections or immunodeficiency (56). In a series of 25 cases (51), 16 (60%) were found to have lymphopenia. Only nine had full immunophenotyping performed and two of these had a picture of cDGS. A further five of eight patients dying in infancy had marked lymphopenia but did not have lymphocyte phenotyping performed so it is possible that the incidence of cDGS was higher. The authors do however concede that this series of patients referred to a specialist center might present a biased view. Nevertheless, the proportion of children with CHARGE syndrome affected by a significant immunodeficiency is probably at least as high as the proportion in DGS associated with 22q deletion. This conclusion would be consistent with the report of a series of 54 cases of patients referred for thymus transplantation for cDGS where the numbers of CHARGE and of 22q deleted cases were roughly in proportion to the incidences of the two genetic defects (55).

### Immunodeficiency in partial DGS

The majority of children with thymic insufficiency as part of DGS, whatever the underlying cause, will have only a partial form of immunodeficiency. The consequences are an increased susceptibility to infections and sometimes immunodysregulation resulting in autoimmunity. A wide range of T-cell immunity is seen in pDGS from near normal to near completely deficient. Normal or near normal T-cell numbers can be found even in those with an apparently absent or hypoplastic thymus and in these it is probable that some thymic tissue is ectopically placed (57). There may be a small subset of more severely deficient 22q.11 – pDGS patients with T-cell numbers near the lower end of the range who have an increased susceptibility to “T-cell” type pathogens such as *Candida albicans* and viral infections and an increased non-cardiac mortality (58, 59). Hypocalcemia was an associated feature of this subgroup in one of these studies (58) and was also associated with lymphopenia in another study of CHARGE patients (51). Otherwise there is no correlation between the severity of immunodeficiency and the clinical phenotype in regard the other features of DGS (60). Most pDGS patients do not suffer opportunistic or life-threatening infections. Their infections tend to be of a sinopulmonary nature, more consistent with a humoral than a T-cell immunodeficiency. Susceptibly to such respiratory tract infections is likely to be at least partly due to non-immunological issues such as velo-pharyngeal insufficiency, eustachian tube dysfunction, disco-ordinate swallowing, gastro-esophageal reflux, and sometimes tracheo-bronchomalacia (59, 61).

As is the case with other partial T-cell deficient states, autoimmune disease can occur in pDGS. This has most commonly been reported as manifesting with immune cytopenias, arthritis, or hyper/hypothyroidism (62–73). The mechanism by which tolerance breaks down leading to autoimmunity in pDGS is not clear. Many forms of primary immunodeficiency are associated with an increased risk of autoimmune disease including conditions not associated with dysregulation of T cells. It has been suggested that persistent antigen stimulation from frequent and/or persistent infections may predispose to autoimmunity (74). However, in pDGS autoimmunity is not predominantly found in those with the most severe or frequent infections (65, 75). It is more likely that disturbance of central or peripheral tolerance or both occur as a consequence of the thymic abnormality. In the normal situation, central tolerance is generated through the presentation of tissue specific peptides to developing thymocytes by medullary thymic epithelial cells in the context of autologous major histocompatibility antigens and under the regulation of the autoimmune regulator (AIRE). There is subsequent deletion (negative selection) of thymocytes recognizing these self-antigens. It is possible that a reduced bulk of thymic tissue in pDGS results in incomplete negative selection or that AIRE expression in pDGS is reduced or otherwise abnormal. The author is not aware of any reported studies of AIRE expression in thymic tissue from pDGS cases. Abnormalities of thymic tissue, including AIRE expression, has been described in SCID due to recombination activating gene (RAG) defects and may contribute to the multisystem inflammation/autoimmunity seen in Omenn syndrome (76) though these patients also have a defect of regulatory T cells suggesting a possible peripheral tolerance defect in addition (77). In pDGS, negative selection must occur in relation to most antigens since the autoimmune disease seen is usually limited to one or two organs or systems. By contrast, in autoimmune polyglandular syndrome type 1 (APS-1) (78) caused by mutations in the AIRE gene, multiple autoimmune disorders are typical. Breakdown of peripheral tolerance is another possible explanation for autoimmunity in pDGS. One study reported reduced numbers of circulating CD4+ Foxp3+ T cells, described as natural T regulatory cells (nTregs) in pDGS patients compared to controls. The levels of these cells correlated closely with the numbers of recent thymic emigrant cells suggesting they were at least partially thymus derived (75). Another study (79) looked at CD4+ CD25+ cells which include Treg cells. In both studies these populations were present in reduced numbers in pDGS patients compared to controls at all ages but there was no difference between the levels in patients with and without autoimmunity. Immunological assessment of pDGS patients often shows low overall numbers of T cells compared to normal with a tendency to improve after the first year of life, although in 10p deletion syndrome a progressive T-cell lymphopenia has been reported (33). Mitogen responsiveness is generally normal in pDGS (80, 81). An increase in T-cell numbers with age may in part be due to the development of oligoclonal expansions resulting in abnormal T-cell receptor spectratypes. (75, 82–85). Naïve T-cell proportions are lower than normal and fall off more quickly with age than in an age – matched control group (82). T-cell recombination excision circles (TRECs) were found to correlate well with the proportions of circulating naïve T cells (86), though a cautionary note was struck by the report of a patient, with what turned out to be pDGS, showing very low TREC levels with good naïve cell proportions (87).

Humoral immune defects and disturbance of B-cell immunity were recognized very early on after DGS was first described (50). These may be relevant to the types of infections suffered. A number of relatively small series have looked at immunoglobulin and antibody levels in DGS associated with 22q.11 deletion (62, 63, 65, 68, 75, 88–90) and CHARGE syndrome (51). Low immunoglobulin levels were reported with variable frequency, most commonly affecting IgM but also occasionally causing a sufficiently low IgG to merit immunoglobulin replacement therapy. Defective antibody responses to polysaccharide antigens were reported in a significant minority of patients. A recently published, much larger study reported on over 1000 patients, with a median age of 3 years, from the European Society for Immunodeficiency and US Immunodeficiency Network (91). Forty two percent were recorded as having 22q.11 deletion but the underlying cause was not reported in the remainder. Overall, 2.7% were on immunoglobulin replacement therapy (3% in those over 3 years old). In the over 3 years age group 6.2% had IgG levels below 5 g/l. Amongst patients over 3 years of age, around 0.7% had complete and 1% partial IgA deficiency whilst 23% had low levels of IgM. There was no association between low immunoglobulin levels, in any of the isotypes, and T-cell counts nor between low T-cell counts and immunoglobulin levels. The authors acknowledged that the data were incomplete and that there may have been some reporting bias in that these patients were registered through immunodeficiency networks. Nevertheless, this study provides the best estimate of the prevalence of humoral immune deficit in DGS. B-cell numbers were not reported in this study but in another study were found to be generally normal though sometimes low in the first year of life, normalizing later (92). The repertoire of IgH usage is also normal but further diversification through somatic hypermutation is deficient (93). It has also been shown that the maturation of B-cells toward a memory phenotype is impaired in pDGS (88). Given the specific role of the thymus in T- but not B-cell development it is probable, but not proven, that B-cell abnormalities are secondary to the T-cell deficiency in these patients.

### Immunodeficiency in complete DGS

Complete DGS is associated with athymia and results in a picture of SCID in a patient showing other variable features of DGS. Affected patients suffer opportunistic infections and, like other infants with SCID, are likely to die early unless they can be treated with a corrective procedure. In addition to susceptibility to infections these patients are at risk from transfusion acquired graft versus host disease (55).

**Figure 1:**
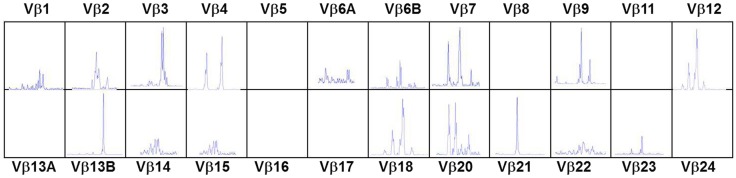
**T-cell receptor spectratyping of 24 Vβ families obtained using polymerase chain reaction amplification across the VDJ region and then plotting according to the size of the PCR products. Patient with atypical cDGS showing very abnormal spectratype with several completely missing families and abnormal skewed distribution in other families**.

**Figure 2:**
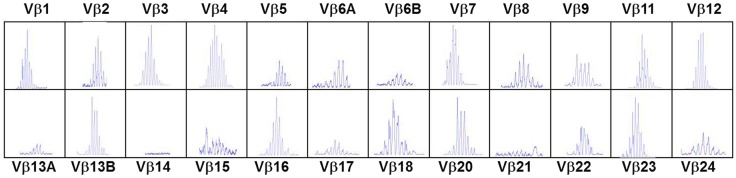
**T-cell receptor spectratyping performed as in legend to Figure 1. Same patient as in Figure 1, 23 months after thymus transplantation. Much more normal spectratype. All families represented mostly with Gaussian distribution**.

In the typical form of cDGS the T-cell numbers are <50/cumm and mitogen responses are absent. B cells are usually present in normal numbers and NK cells in normal or high numbers. In a proportion of cases there may be some mature T cells present either through maternal engraftment (94) or through oligoclonal expansion of memory phenotype T cells which have developed without thymic processing (95). In the latter case, as in SCID these cells can mediate severe inflammation leading to an Omenn-like picture with erythrodermic rashes, enteropathy, and lymphadenopathy (53, 96) This is called atypical cDGS. The diagnosis of complete athymia then depends on showing absence (<50/cumm) of T cells with a naïve (CD3 + CD45 RA+CD62L+) phenotype as well as abnormal T-cell receptor usage either by T-cell receptor spectratyping or FACS analysis of usage of V Beta TCR chains (96). An example of the abnormal spectratype in an atypical cDGS patient is shown in Figure 1 which can be compared to the normal spectratype achieved in the same patient after successful thymus transplantation (Figure 2). Mitogen responsiveness is usually, but not invariably, impaired in these atypical patients (96).

Diagnosis of cDGS depends on the findings of the clinical features of DGS together with the above immunological findings with or without identification of one of the associated genetic abnormalities. A recent report (97) describes two patients with absent T cells and DGS associated with 22q.11 deletion who were also found to have pathogenic mutations in the DCLRE1C (Artemis) gene, a classical cause of SCID. A clue to the latter diagnosis was the virtual absence of B cells as well as T cells which is very unusual in cDGS alone.

Newborn screening for SCID using TREC detection on blood spots has been in place in certain states of USA for around 3 years (98, 99). Since TRECs will be absent or extremely low (86) this allows the early diagnosis of cDGS. In the California program (98) screening of nearly one million newborns picked up one cDGS case who went on to thymus transplantation, eight with T-cell lymphopenia associated with 22q.11 deletion and one with CHARGE association. Picking up the latter group was useful in the early identification of these children as having significant immunodeficiency and allowed infection prevention measures to be put in place including avoidance of live viral vaccinations. Newborn screening programs should offer the opportunity of a better outcome through earlier intervention in both cDGS and some cases of pDGS.

## Corrective Treatment for cDGS

### Hematopoietic cell transplantation

Treatment with hematopoietic cell transplantation (HCT) for athymia is dependent on the transfer of mature post-thymic T cells. Long term survival after such transplants has been reported (100, 101) though at a low rate (41–48%) compared to survival after HCT for SCID (102). Survival in the subgroup receiving matched sibling donor transplants was better at over 60% (100). Mortality was related to other features of DGS, to graft versus host disease and to pre-existing viral infections. The quality of immune reconstitution achieved, as expected, is poor with no evidence of naïve T cells and often low CD4 counts with skewed distribution of T-cell receptor usage. However immunoglobulin production and antibody responses were relatively good. Though overall the outcome after HCT for cDGS is not good, in some circumstances, such as overwhelming viral infection, HCT from a matched sibling may be life-saving (103).

### Thymus transplantation

**Figure 3:**
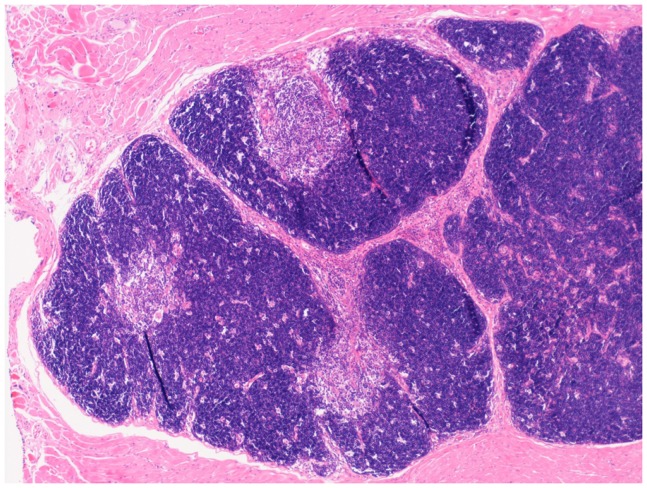
**Low-power view of a biopsy of transplanted thymus stained with Hematoxylin and Eosin. Normal looking thymic tissue surrounded by striated muscle. There is good corticomedullary distinction**.

Replacement of thymic function using allografted tissue was first achieved using human fetal thymic tissue (104, 105). The use of post natal human thymus, necessarily removed at the time of cardiac surgery in infants undergoing median sternotomy, was pioneered by Markert at Duke University (106, 107) and has become established as the treatment of choice for cDGS. More recently this approach has also been used in London using an almost identical approach (manuscript in preparation). The thymus is cultured for 12–21 days prior to transplantation into the quadriceps muscle of the patient. During this period most thymocytes are washed out or undergo apoptosis whilst the thymic stroma is preserved. Patients with atypical cDGS are pre-treated with anti thymocyte globulin and continuing cyclosporine A (108) whilst typical cases receive no pre-conditioning. The results have been published (55, 109) and of 60 patients treated 43 survived (72%). This compares favorably with the outcome after HCT described above though strict comparison is not possible as the thymus transplant patients were a selected group. After successful transplantation, patients develop host derived naïve T cells with a normal T-cell receptor repertoire (Figure 2), normal mitogen responses and antigen specific immune responses restricted to the host major histocompatibility complex (MHC). There is normalization of the TCR repertoire in circulating regulatory T cells (110). Biopsies of transplanted thymus taken from 2 months onward show thymopoiesis (111) and normal thymus architecture (Figure 3). The levels of circulating T cells achieved do not usually match normal age matched controls and are more akin to the levels seen in children with pDGS. Tolerance to the donor’s MHC has been demonstrated (112) and this has been exploited to enable parathyroid transplantation from a parent in situations where there is coincidental partial MHC class 2 matching between the donor and the parent (113).

Deaths after thymus transplantation were related mainly to pre-existing co-morbidities, mostly chronic lung disease and systemic viral infections such as cytomegalovirus (CMV) (114). This virus is a particular problem. Screening of potential thymic donors always excludes CMV positive donors but a proportion of cDGS patients will have acquired the virus before thymus transplantation. Biopsies of transplanted thymus tissue from two patients with CMV in the Markert series showed no evidence of thymopoiesis even though the epithelium was viable (111). Both patients died. A similar appearance was found in a CMV infected patient in London who also died without evidence of thymopoiesis (manuscript in preparation). The mechanism by which CMV interferes with thymopoiesis is not clear but as a result of this experience, CMV infection should be considered at least a relative contraindication to thymus transplantation. After successful thymus transplantation patients are able to control infections and to come off antibiotic prophylaxis and immunoglobulin therapy with normal responses to immunization. The main problem that has been encountered is the development of autoimmunity. Around one third of patients have shown autoimmunity, mainly hypothyroidism but also with a significant number of immune cytopenias (109). It is interesting that this spectrum of autoimmunity is similar to that seen in pDGS patients, as discussed above, and may have the same causation or may be related to faulty thymic education related to the fact that the transplanted thymic epithelial cells are not MHC matched, as discussed below. No clinical or methodological correlates with risk of autoimmune development could be identified in the Duke University series. (114).

The success of transplantation of thymus which is not matched at the MHC loci offers interesting insights into thymocyte development. In particular, it suggests that positive and negative selection of developing thymocytes can occur in the absence of self MHC expressed on thymic epithelial cells. The mechanism by which this takes place is incompletely understood. Reconstitution experiments in nude mice with MHC incompatible thymic tissue showed that functional T cell development could be supported by haematopoeitic cell-expressed MHC instead of TEC- expressed MHC (115). Further work showed that development of functional CD4 (but not CD8) cells however does seem to require interaction with MHC on TECs but not any particular allelic form of MHC (116). Under the influence of AIRE expressed on thymic epithelium dendritic cells have been shown to have a role in negative selection in mice (117). Whilst negative selection may be imperfect resulting in autoimmunity in some cases, it must be largely effective since multiple system/organ autoimmunity from widespread lack of central tolerance has not been seen. Positive selection has also been shown to be mediated by fibroblasts (118) and by thymocytes (119, 120). Influx of these cell types expressing host MHC to the developing thymus allograft could therefore have the potential for mediating the selection processes.

## Conclusion

Study of the thymic deficiency in DGS provides insights into the development of the thymus and the mechanisms of thymopoiesis required to generate a robust and diverse T-cell mediated immunity. Thymus transplantation offers a novel way of correcting the immunodeficiency in this disorder.

## Conflict of Interest Statement

The author declares that the research was conducted in the absence of any commercial or financial relationships that could be construed as a potential conflict of interest.
